# Factors associated with health professionals’ stress reactions, job satisfaction, intention to leave and health-related outcomes in acute care, rehabilitation and psychiatric hospitals, nursing homes and home care organisations

**DOI:** 10.1186/s12913-024-10718-5

**Published:** 2024-03-02

**Authors:** Karin Anne Peter, Christian Voirol, Stefan Kunz, Andrea Gurtner, Fabienne Renggli, Typhaine Juvet, Christoph Golz

**Affiliations:** 1 Department of Health Professions, Bern University of Applied Sciences, Bern, Switzerland; 2 Haute Ecole Arc Santé, University of Applied Sciences and Arts Western Switzerland, Neuchatel, Switzerland; 3 Department of Psychology, University of Montreal, Montreal, Canada; 4 Department of Medicine, University of Montreal, Montreal, Canada; 5 Department of Business Economics, Health and Social Care, University of Applied Sciences and Arts of Southern Switzerland, Lugano, Switzerland; 6 Institute New Work, Department of Business School, Bern University of Applied Sciences, Bern, Switzerland

**Keywords:** Work-related stress, Intention to leave, Job satisfaction, Hospital, Nursing home, Home care organization, Health professionals

## Abstract

**Abstract:**

The aim of this study is to identify (1) the extent of work-related stress and (2) stressors associated with cognitive and behavioral stress reactions, burnout symptoms, health status, quality of sleep, job satisfaction, and intention to leave the organization and the profession among health professionals working in acute care /rehabilitation hospitals, psychiatric hospitals, nursing homes, and home care organizations.

**Background:**

Health professionals are faced with various stressors at work and as a consequence are leaving their profession prematurely. This study aimed to identify the extent of work-related stress and stressors associated with stress reactions, job satisfaction, and intention to leave and health-related outcomes among health professionals working in different healthcare sectors (acute care, rehabilitation and psychiatric hospitals, nursing homes and home care organizations).

**Methods:**

This study is based on a repeated cross-sectional design, which includes three data measures between 2017 and 2020 and 19,340 participating health professionals from 26 acute care / rehabilitation hospitals, 12 psychiatric hospitals, 86 nursing homes and 41 home care organizations in Switzerland. For data analysis, hierarchical multilevel models (using AIC) were calculated separately for hospitals, nursing homes, and home care organizations, regarding health professionals’ stress symptoms, job satisfaction, intention to leave the organization / profession, general health status, burnout symptoms, and quality of sleep.

**Results:**

The main findings reveal that the incompatibility of health professionals’ work and private life was significantly associated (*p* < 0.05) with their stress reactions, job satisfaction, intention to leave, and health-related outcomes in all the included work areas. The direct supervisor’s good leadership qualities were also associated with health professionals’ job satisfaction regarding all work areas (B ≥ 0.22, *p* = 0.000). In addition, a positive perceived bond with the organization (B ≥ 0.13, *p* < 0.01) and better development opportunities (B ≥ 0.05, *p* < 0.05) were associated with higher job satisfaction and a lower intention to leave the organization and profession among health professionals. Also, a younger age of health professionals was associated with a higher intention to leave the organization and the profession prematurely in all the included work areas. High physical (B ≥ 0.04, *p* < 0.05) and quantitative demands (B ≥ 0.05, *p* = 0.000) at work were also associated with negative health-related outcomes.

## Introduction

Around the globe, healthcare systems are struggling with a shortage of health professionals and a potential destabilization of the quality and availability of care provided [1]. Work-related stress and poor working conditions are among the main reasons why health professionals leave their profession prematurely [2–4]. As recent studies show, the COVID-19 pandemic has further exacerbated the problem of stress and poor working conditions among health professionals in various countries and work areas [5–7].

Work-related stress can be defined as “a pattern of reactions that occur when workers are confronted with demands or pressures that are not matched to their knowledge, abilities and skills, and which challenge their ability to cope” [8, 9]. The model of ‘causes and consequences of work-related stress’ [8, 10] is the underlying theoretical background of this study. It explains the causes of stress (stressors), stress reactions (short-term), and consequences of work-related stress (long-term) on the employee as well as their inter-reactions (stressors are associated with stress reactions and long-term consequences) [8, 10]. Stressors at work are particularly pronounced in the daily work of health professionals, such as high emotional demands due to confrontation with sickness and death, or aggression at work, or high physical demands when lifting or moving patients [11–13]. Working under time pressure, doing overtime, long working hours, and understaffing are also well-known stressors among health professionals [14–16]. In addition, they are confronted with a lack of opportunities for development, poor leadership qualities of superiors and a high exposure to infectious disease or hazardous substances in their daily work [4, 17]. Furthermore, they are strongly affected by incompatibilities of work and private life, shift work, and problems with demarcation between work and leisure time [4, 18–20]. As previous studies indicate, a high level of stressors at work is associated with health professionals’ increasing anxiety, depression, job dissatisfaction, and the intention to leave their profession prematurely [21, 22].

There are multiple studies regarding stressors, stress reactions, and long-term consequences among health professionals working in different management levels [23, 24], professional roles [2, 3, 21, 25], or work areas [14, 26, 27]. However, most studies focus on one specific work area (e.g., acute care hospitals or nursing homes) [3, 15, 19] or on only one specific health profession (e.g., nurses) [14, 28]. Thus, studies with a focus on work-related stress among health professionals combining and comparing different work areas in the healthcare sector are rare.

Therefore, the aim of this study is to identify (1) the extent as well as differences of work-related stress in various work areas and (2) stressors associated with cognitive and behavioral stress reactions, burnout symptoms, health status, quality of sleep, job satisfaction, and intention to leave the organization and the profession among health professionals working in acute care /rehabilitation hospitals, psychiatric hospitals, nursing homes, and home care organizations.

## Methods

### Design

This study presents the results of the national STRAIN project ‘work-related **str**ess **a**mong health professionals **in** Switzerland’ [4, 23]. These results are based on a repeated cross-sectional design, using three measurements between September 2017 – March 2018 (T^0^), January – April 2019 (T^1^), and March – September 2020 (T^2^), conducted among Swiss health professionals working in acute care / rehabilitation hospitals, psychiatric hospitals, nursing homes, and home care organizations. Only 4% of participants took part in all three measurement points. Participating organizations were free to choose the time for data collection when it suited them best.

### Recruitment and study sample

For recruitment, potential healthcare organizations were randomly selected from a register (Swiss Federal Statistical Office in 2016) of all hospitals, nursing homes, and home care organizations in Switzerland. We excluded organizations that were too small in size (average number of beds < 20, fewer than 7 employees) or that were specialized (e.g., in neonatology) [23]. The randomization process was computer-based using randomizer.org and considered a geographically representative sample for Switzerland (69% Swiss or Standard German-speaking, 23% French-speaking, 8% Italian-speaking). Thereby, 100 hospitals, 100 nursing homes, and 100 home care organizations were randomly selected and invited to participate in the study. The invited organizations received information about the study using a study flyer and a short film. In the end, a total of 26 acute care / rehabilitation hospitals, 12 psychiatric hospitals, 86 nursing homes, and 41 home care organizations (117 German-speaking, 39 French-speaking, 9 Italian-speaking) took part in the study.

### Data collection

For the data collection, a contact person in each participating organization was responsible for distributing the questionnaire. The questionnaire was distributed to all healthcare professionals working in the organization at the time of data collection. Nurses, midwives, physicians, medical-technical professionals, medical-therapeutic professionals, and employees from the administration and research at all hierarchies and skill levels (e.g., health professionals in training) were included in the study. The questionnaire was available in German, French, and Italian in two online versions (Surveymonkey®, UmfrageOnline®), as well as in a paper version. Participating health professionals had one month to complete the questionnaire and received a reminder after the first two weeks during the data collection period. The data collection was on a voluntary basis for all participating organizations as well as all participating health professionals within them.

### STRAIN – questionnaire

The study used the STRAIN questionnaire, which is designed based on the model of ‘causes and consequences of work-related stress’ [8, 10] and, therefore, is composed of scales assessing stressors at work (e.g., demands at work, work–private life conflict), stress reactions (behavioral and cognitive stress reactions) and long-term consequences (e.g., burnout symptoms, job satisfaction, general health status, quality of sleep, intention to leave the organization or profession). The STRAIN questionnaire consists of widely used, valid, and reliable scales (e.g., on quantitative demands, influence at work, role clarity) from the Copenhagen Psychosocial Questionnaire (COPSOQ) [29, 30], which is the questionnaire used in the ‘Nurses Early Exit Study’ (NEXT) [31, 32]. The COPSOQ item responses are scored on a five-point Likert scale (to a very large extent, to a large extent, somewhat, to a small extent, to a very small extent, or always, often, sometimes, seldom, never/hardly ever). COPSOQ scale score value ranges from 0 (to a very small extent or never/hardly ever) to 100 (to a very large extent or always). In addition, the 4-item scale on physical demands from the sixth European Working Conditions Survey – EWCS [33] (7-point Likert scale) and the self-rated general health status on a range from 0 (worst health, you can imagine) to 100 (best health, you can imagine) (using EQ-5D-5L [34] were included. Further details on the STRAIN questionnaire were published previously [4, 36, 37].

### Data analysis

Data were analyzed using SPSS 25® and R Studio 4.2.2 [37]. According to the original author, all items from the COPSOQ, EWCS and NEXT were transformed to a value range from 0 (minimum value) to 100 points (maximum value) [29, 33]. If less than half of the questions in a scale were answered, no average score was calculated [29].

In the first step, we calculated the descriptive statistics describing the study sample divided into the included work areas (hospitals, nursing homes, home care organization).

In the second step, we calculated the extent of stressors, stress reactions, and long-term consequences among health professionals working in acute care/ rehabilitation hospitals, psychiatric hospitals, nursing homes, and home care organizations and tested for significant differences using the Kruskal–Wallis H test (significance level of 0.05, using the Bonferroni correction for multiple tests) as well as a pairwise comparison (Dunn-Bonferroni tests), since the test of homogeneity of variance was significant. There were no equal-sized samples of data.

**Fig. 1:**
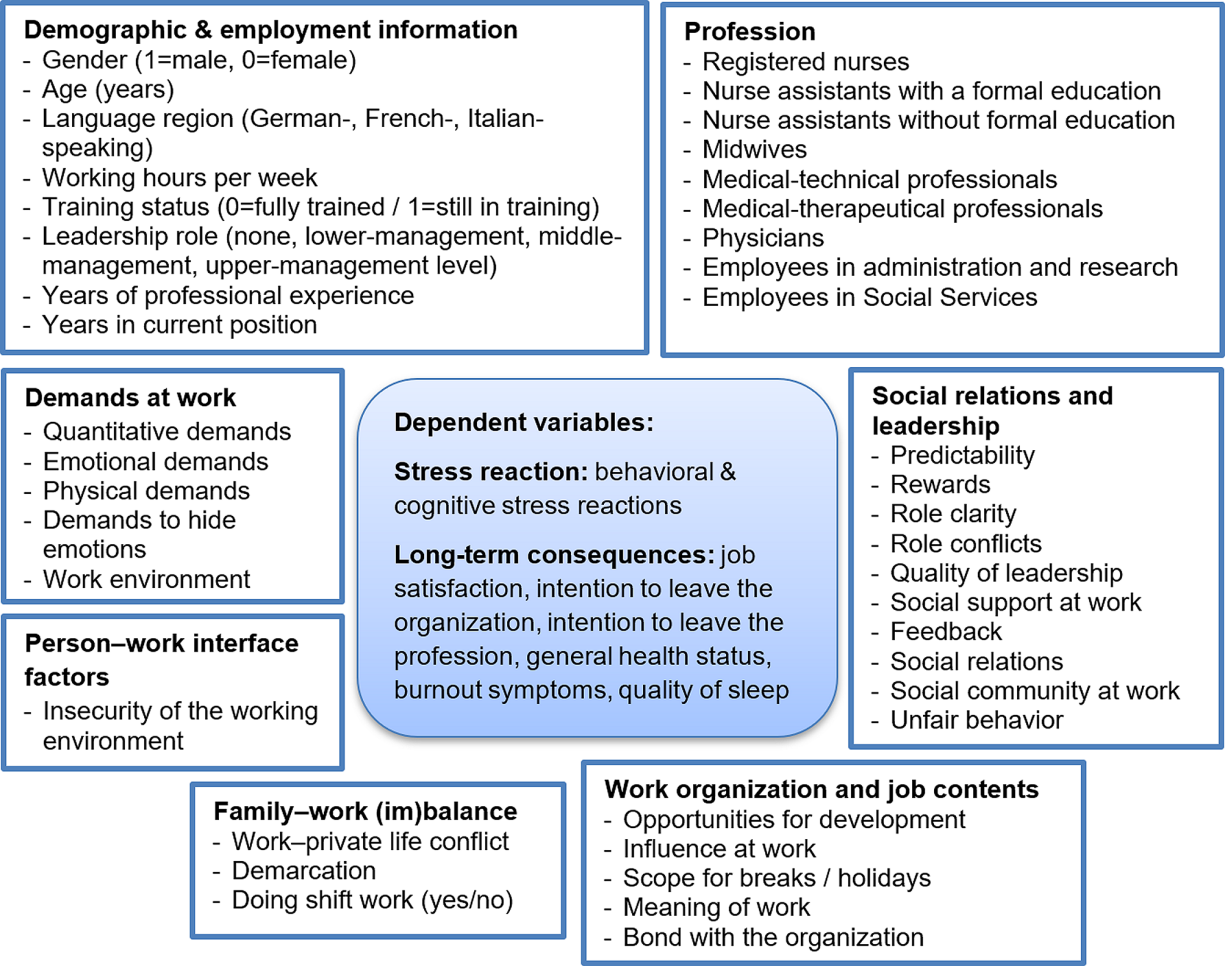
Dependent and independent variables in the regression models

In the third step, we calculated separate hierarchical multiple regression models for (1) acute care /rehabilitation hospitals, (2) psychiatric hospitals, (3) nursing homes, and (4) home care organizations using health professionals’ data’ (level 1) nested in organizations (level 2). Regression models were calculated for the following outcome variables: behavioral stress symptoms; cognitive stress symptoms; job satisfaction; intention to leave the organization; intention to leave the profession; general health status; burnout symptoms; and quality of sleep. All the independent variables included in the regression models are presented in Fig. 1. A backward model selection with the MASS package was conducted with Akaike Information Criterion [38]. The models were then fitted using the lme4 package. We computed standardized and nonstandardized beta coefficients, *p*-values, standard errors, CI, and R-squared (marginal / conditional) [39, 40]. Since the assumption of heteroskedasticity was not met for the models, standard errors, *p*-values, and CI were bootstrapped (*r* = 999, bias corrected and accelerated, 95% CI).

## Results

### Study sample description

A total of 19,340 health professionals took part in the study. Participants were mainly women (83%) and from the German-speaking part of Switzerland (83% German-speaking, 15% French-speaking, 2% Italian-speaking). The study sample included registered nurses (48%), nurse assistants (28%), midwives (1%), medical-technical (3%), medical-therapeutic professionals (9%), physicians (7%), employees in administration and research (3%), and social services (2%). Most participants had no management responsibilities (84%), 11% worked in a lower, 4% in a middle, and 2% in an upper management position. Table 1 provides further details on the distribution of sex, language region, profession, and leadership positions of the participants working in acute care / rehabilitation hospitals, psychiatric hospitals, nursing home, and home care organizations.

**Table 1:** Descriptive statistics regarding different healthcare settings

		All settings *n* = 19,340		Acute care and rehabilitation hospitals *n* = 8179		Psychiatric hospitals *n* = 4464		Nursing homes *n* = 3860		Home care organizations *n* = 3860	
		*n*	**%**	*n*	%	*n*	%	*n*	%	*n*	%
**Sex**	Women	**15,816**	**83%**	6690	83%	3150	72%	3320	87%	2656	95%
	Men	**3205**	**17%**	1366	17%	1216	28%	485	13%	138	5%
**Language region**	German-speaking	**14,871**	**83%**	6301	80%	4124	93%	2361	76%	2085	82%
	French-speaking	**2715**	**15%**	1609	20%	101	2%	608	20%	397	16%
	Italian-speaking	**406**	**2%**	0	0%	213	5%	146	5%	47	2%
**Profession**	Registered nurses	**8185**	**48%**	4086	56%	1901	50%	929	27%	1269	52%
	Nurse assistants with a formal education	**3537**	**21%**	831	11%	348	9%	1588	46%	770	32%
	Nurse assistants without formal education	**1228**	**7%**	94	1%	47	1%	734	21%	353	14%
	Midwives	**170**	**1%**	170	2%	0	0%	0	0%	0	0%
	Medical-technical professionals	**523**	**3%**	523	7%	0	0%	0	0%	0	0%
	Medical-therapeutical professionals	**1509**	**9%**	656	9%	694	18%	159	5%	0	0%
	Physicians	**1101**	**7%**	649	9%	413	11%	39	1%	0	0%
	Employees in administration and research	**440**	**3%**	269	4%	100	3%	16	1%	55	2%
	Employees in Social Services	**353**	**2%**	31	0%	296	8%	26	1%	0	0%
**Leadership position**	Upper-management level	**280**	**2%**	98	1%	79	2%	30	1%	73	3%
	Middle-management level	**706**	**4%**	269	4%	266	6%	110	3%	61	2%
	Lower-management level	**1945**	**11%**	800	10%	467	11%	453	13%	225	8%
	Without management responsibilities	**15,295**	**84%**	6601	85%	3361	81%	2952	83%	2381	87%

*n* = number of cases

### Extent of work-related stress in different work areas

Table 2 shows an overview of the extent of various stressors, stress reactions, and long-term consequences among health professionals working in different work areas in the healthcare sector (hospitals, nursing homes, home care organizations).

**Table 2:** Extent of work stressors, stress reaction and long-term consequences among different healthcare settings

	All settings (*n* = 19,340)			1 = Acute care/rehabilitation hospitals (*n* = 8197)		2 = Psychiatric hospitals (*n* = 4464)		3 = Nursing homes (*n* = 3860)		4 = Home care organizations (*n* = 2837)		Kruskal–Wallis test	
	*N*	Mean	SD	Mean	SD	Mean	SD	Mean	SD	Mean	SD	*p*-value	*Significant differences between settings (pairwise comparison)
**Stressors at work**													
Quantitative demands	18,098	54.5	17.3	**59.3**	15.5	51.7	17.1	52.0	17.8	48.4	17.8	0.000	1vs2; 1vs3; 1vs4; 2vs3; 2vs4
Sensorial demands	18,089	83.1	14.6	**86.2**	13.5	75.6	15.5	84.0	13.8	84.6	13.3	0.000	1vs2; 1vs3; 1vs4; 2vs3; 2vs4
Emotional demands	18,038	60.5	15.5	59.3	15.8	59.7	13.7	**68.3**	15.6	54.3	12.9	0.000	1vs3; 1vs4; 2vs3; 2vs4; 3vs4
Physical demands	17,975	36.5	22.6	40.9	21.9	18.6	14.1	**46.8**	23.5	37.3	18.4	0.000	1vs2; 1vs3; 1vs4; 2vs3; 2vs4; 3vs4
Demands to hide emotions	17,239	39.6	22.7	42.2	22.4	41.4	21.6	35.6	23.7	36.0	22.4	0.000	1vs3; 1vs4; 2vs3; 2vs4
Work environment	17,947	33.3	19.4	**38.4**	19.5	27.7	18.9	29.8	19.0	32.4	17.0	0.000	1vs2; 1vs3; 1vs4; 2vs3; 2vs4; 3vs4
Opportunities for development	18,166	71.0	15.5	72.3	15.1	71.8	15.3	68.7	16.8	69.7	15.0	0.000	1vs3; 1vs4; 2vs3; 2vs4
Influence at work	17,876	51.5	20.2	**48.0**	20.1	58.1	17.9	51.2	21.0	51.5	20.3	0.000	1vs2; 1vs3; 1vs4; 2vs3; 2vs4
Scope for breaks / holidays	17,831	61.1	20.8	57.9	20.0	66.0	19.7	56.6	22.3	69.0	18.8	0.000	1vs2; 1vs4; 2vs3; 2vs4; 3vs4
Meaning of work	18,064	83.5	16.1	82.5	16.1	**79.3**	17.4	87.4	14.8	87.2	14.0	0.000	1vs2; 1vs3; 1vs4; 2vs3; 2vs4
Bond with the organization	17,250	62.7	20.6	60.6	20.0	60.2	20.2	64.1	21.8	69.8	19.2	0.000	1vs3; 1vs4; 2vs3; 2vs4; 3vs4
Predictability	17,692	63.8	19.8	62.3	19.5	61.5	19.3	65.3	21.1	69.1	18.3	0.000	1vs3; 1vs4; 2vs3; 2vs4; 3vs4
Rewards	17,483	58.6	26.0	**54.3**	26.3	56.7	26.2	62.1	25.1	68.4	22.9	0.000	1vs2; 1vs3; 1vs4; 2vs3; 2vs4; 3vs4
Role clarity	17,805	78.1	14.8	79.2	14.4	**73.4**	15.2	80.3	14.8	79.1	14.1	0.000	1vs2; 1vs3; 2vs3; 2vs4; 3vs4
Role conflicts	17,767	36.0	20.5	**39.3**	20.2	37.6	20.0	33.5	21.6	28.4	18.3	0.000	1vs2; 1vs3; 1vs4; 2vs3; 2vs4; 3vs4
Quality of leadership	17,543	64.5	22.3	**62.7**	22.6	64.0	22.9	65.5	22.1	68.7	19.8	0.000	1vs2; 1vs3; 1vs4; 2vs4; 3vs4
Social support at work	17,460	77.1	17.1	**75.6**	16.9	78.3	17.0	76.6	17.8	80.5	16.5	0.000	1vs2; 1vs3; 1vs4; 2vs3; 2vs4; 3vs4
Feedback	17,334	50.5	20.8	**49.3**	20.9	51.1	19.9	53.2	21.3	**49.3**	20.7	0.000	1vs2; 1vs3; 2vs3; 2vs4; 3vs4
Social relations at work	17,375	58.0	27.2	63.6	23.6	60.7	25.8	59.8	25.2	**36.0**	30.1	0.000	1vs2; 1vs3; 1vs4; 2vs4; 3vs4
Social community at work	17,364	79.9	14.7	79.4	14.3	80.1	14.8	78.5	15.7	82.7	13.7	0.000	1vs4; 2vs3; 2vs4; 3vs4
Unfair behavior	17,138	13.7	21.4	14.6	21.6	12.3	20.4	**16.9**	23.6	9.2	17.6	0.000	1vs2; 1vs3; 1vs4; 2vs3; 2vs4; 3vs4
Insecurity of the working environment	17,793	28.4	25.1	**31.6**	25.7	25.0	22.9	29.6	26.7	23.1	22.5	0.000	1vs2; 1vs3; 1vs4; 2vs3; 2vs4; 3vs4
Work–private life conflict	17,441	28.1	20.9	**30.8**	21.4	27.6	20.0	27.2	21.6	22.6	18.4	0.000	1vs2; 1vs3; 1vs4; 2vs4; 3vs4
Demarcation	17,329	32.2	22.1	**34.7**	22.0	29.0	21.3	32.7	21.9	29.3	22.4	0.000	1vs2; 1vs3; 1vs4; 2vs4; 3vs4
**Stress reactions**													
Behavioral stress symptoms	17,186	29.9	21.8	31.2	22.5	30.2	20.8	29.4	22.3	26.8	20.5	0.000	1vs3; 1vs4; 2vs4; 3vs4
Cognitive stress symptoms	17,239	26.7	19.5	**28.0**	19.8	25.5	19.0	26.2	20.2	25.5	18.1	0.000	1vs2; 1vs3; 1vs4
**Long-term consequences**													
Job satisfaction	17,330	70.4	14.7	**69.2**	14.8	71.1	14.9	**69.9**	14.7	73.0	13.5	0.000	1vs2; 1vs4; 2vs3; 2vs4; 3vs4
Intention to leave the organization	17,272	20.2	22.7	**22.0**	23.2	**22.5**	22.9	17.9	22.8	15.4	20.2	0.000	1vs3; 1vs4; 2vs3; 2vs4; 3vs4
Intention to leave the profession	17,269	16.1	21.3	**17.5**	21.9	15.9	20.6	15.4	21.7	13.8	19.5	0.000	1vs2; 1vs3; 1vs4; 2vs4;
General health status	15,674	79.0	17.0	80.0	17.1	78.3	16.7	77.7	17.5	79.5	15.8	0.000	1vs2; 1vs3; 1vs4; 3vs4
Burnout symptoms	17,116	42.2	20.9	**43.6**	20.9	40.6	19.5	**42.8**	22.7	40.1	20.0	0.000	1vs2; 1vs4; 2vs3; 3vs4
Quality of sleep	17,110	68.8	19.2	**66.8**	19.1	69.8	19.1	69.0	19.6	72.2	18.2	0.000	1vs2; 1vs3; 1vs4; 2vs4; 3vs4

All scales are scored from 0 (minimum value) to 100 (maximum value), *N* = number of cases in total, M = mean, SD = standard deviation, *pairwise comparison using the significance level of 0.05 (2-sided), adjusted by the Bonferroni correction for multiple tests, highest / lowest scores are highlighted

#### Extent of various stressors at work

The results on various work stressors shows that health professionals working in acute care and rehabilitation hospitals reported the highest quantitative (e.g., work at a high pace, doing overtime) demands (M = 59.3; SD = 15.5), and sensorial (e.g., precision, vision, attention) demands (M = 86.2; SD = 13.5) at work. Health professionals working in acute care and rehabilitation hospitals also reported having the most demanding work environment (e.g., being exposed to noise, cold, chemicals) (M = 38.4; SD = 19.5) and the lowest influence at work (e.g., the degree of influence with regard to work) (M = 48.0; SD = 20.1) compared to other work areas. In addition, the perceived rewards (M = 54.3; SD = 26.3) and the quality of leadership of the superior at work (e.g., the superior is good at work planning, solving conflicts) (M = 62.7; SD = 22.6) were lowest among health professionals working in acute care / rehabilitation hospitals. Social support received at work from colleagues or superiors (M = 75.6; SD = 16.9) and feedback (M = 49.3, SD = 20.9) was also lowest, while they reported the highest scores on role conflicts due to contradicting role requirements at work (M = 39.2; SD = 20.2). Furthermore, they showed the highest insecurity in terms of the working environment (e.g., due to changes in shift schedules) (M = 31.6, SD = 25.7). In addition, this group reported the highest incompatibility between work and private life (M = 30.8, SD = 21.4) and difficulties with demarcation (e.g., being available in leisure time for work issues) (M = 34.7, SD = 22.0) compared to other work areas.

In psychiatric hospitals, health professionals revealed they have the lowest meaning of work (e.g., perceiving work as meaningful / important) (M = 79.3; SD = 17.4) compared to other work areas. In addition, role clarity (e.g., clear work tasks, objectives, area of responsibility) (M = 73.4; SD = 15.2) was lowest in psychiatric hospitals compared to health professionals working in other work areas.

In nursing homes, health professionals reported the highest emotional (e.g., confrontation with death, aggressive patients) (M = 68.3; SD = 15.6) and physical (e.g., lifting or moving people or heavy loads) (M = 46.8; SD = 23.5) demands at work. In this sector of healthcare, health professionals’ feeling of unfair behavior (e.g., feeling unjustly criticized by colleagues/superior) was also highest (M = 16.9, SD = 23.6) among those working in a nursing home compared to other work areas.

Health professionals working in home care organizations reported receiving less feedback from colleagues / their superior (M = 49.3, SD = 20.7) and to have the lowest social relations at work (e.g., the opportunity to talk to colleagues during work) (M = 36.0; SD = 30.1).

#### Extent of stress reactions and long-term consequences

Health professionals working in acute hospital or rehabilitation hospitals were most affected by cognitive stress symptoms (e.g., problems concentrating, taking decisions) (M = 28; SD = 19.8), showed higher burnout symptoms (M = 43.59; SD = 20.87), and had lower quality sleep (M = 66.81; SD = 19.12). They also revealed having lower job satisfaction (M = 69.2, SD = 14.8) and the highest intention to leave the organization (M = 17.5; SD = 21.9) or their profession prematurely (M = 17.5, SD = 21.9). Health professionals working in psychiatric hospitals also revealed a high intention to leave the organization (M = 22.5, SD = 22.9). Those health professionals working in nursing homes also showed lower scores regarding their job satisfaction (M = 69.9, SD = 14.7) and higher burnout symptoms (M = 42.8, SD = 22.7).

#### Results of the regression analysis regarding stress reactions

Results of the multiple regression models revealed that an incompatibility of work and private life was associated with increased behavioral stress symptoms (B ≥ 0.45, *p* = 0.000) among health professionals working in all the included areas (acute care, rehabilitation or psychiatric hospitals, nursing homes, or home care organizations, see Table 3). In addition, nurse assistants (with a formal education: B ≥ 0.12, *p* = 0.000 and without a formal education: B ≥ 0.10, *p* = 0.000) working in acute care, rehabilitation hospitals, and home care organizations seemed to be more highly affected in terms of behavioral stress symptoms. High quantitative demands at work were also associated with increased behavioral stress symptoms among health professionals working in acute care, rehabilitation hospitals, and home care organizations (B ≥ 0.13, *p* = 0.000).

**Table 3:** Work stressors associated with health professionals’ stress reactions

	Acute care/rehabilitation hospitals			Psychiatric hospitals			Nursing homes			Home care organizations		
**Outcome variable: behavioral stress symptoms**	Marginal R2 = 0.41; Conditional R2 = 0.42			Marginal R2 = 0.37, Conditional R2 = 0.37			Marginal R2 = 0.46; Conditional R2 = 0.48			Marginal R2 = 0.43; Conditional R2 = 0.45		
	Beta (std.)	SE*	P*	Beta (std.)	SE*	P*	Beta (std.)	SE*	P*	Beta (std.)	SE*	P*
(Intercept)	0.00	4.41	0.000	0.00	0.00	0.560	0.00	6.05	0.000	0.00	4.21	0.000
Age (years)	-0.05	0.04	0.026				-0.11	0.03	0.000	-0.05	0.04	0.028
Years of professional experience				-0.07	0.02	0.001						
Employees in administration and research	-0.04	3.42	0.072				-0.03	6.43	0.124	-0.04	3.32	0.064
Physicians				-0.04	0.02	0.049						
Nurse assistants without formal education	0.10	1.49	0.000							0.10	1.44	0.000
Nurse assistants with a formal education	0.12	0.99	0.000				-0.04	1.07	0.056	0.12	1.05	0.000
Registered nurses				-0.03	0.02	0.124	-0.03	1.22	0.180			
Working hours per week				0.08	0.02	0.000						
Gender				-0.06	0.02	0.007						
Emotional demands at work							0.04	0.03	0.078			
Physical demands at work				0.08	0.02	0.000						
Quantitative demands at work	0.13	0.03	0.000	0.03	0.02	0.129	0.04	0.03	0.086	0.13	0.03	0.000
Demands to hide emotions	0.06	0.02	0.004	0.05	0.02	0.014	0.11	0.02	0.000	0.06	0.02	0.008
Meaning of work				-0.04	0.02	0.072	-0.08	0.03	0.000			
Opportunities for development	-0.05	0.03	0.038	-0.05	0.03	0.033				-0.05	0.03	0.022
Influence at work				-0.04	0.02	0.073						
Bond with the organization	-0.08	0.03	0.000	-0.07	0.02	0.001	-0.07	0.02	0.000	-0.08	0.03	0.002
Quality of leadership				0.05	0.02	0.051						
Feedback				-0.05	0.02	0.025						
Social community at work							-0.09	0.03	0.000			
Role clarity				-0.05	0.02	0.015						
Social relations at work	-0.04	0.02	0.110	0.04	0.02	0.046				-0.04	0.02	0.112
Unfair behavior				0.05	0.02	0.020						
Social support at work	-0.09	0.03	0.002				0.04	0.03	0.100	-0.09	0.03	0.000
Predictability							-0.07	0.03	0.006			
Insecurity of the working environment	0.06	0.02	0.038				0.04	0.02	0.076	0.06	0.02	0.030
Difficulties with demarcation							0.04	0.02	0.040			
Work–private life conflict	0.46	0.03	0.000	0.45	0.02	0.000	0.45	0.03	0.000	0.46	0.03	0.000
Doing shift work				-0.08	0.02	0.001	-0.04	1.21	0.070			
Language region: German-speaking							-0.06	3.21	0.268			
Language region: French-speaking							-0.11	3.51	0.040			
**Outcome variable: cognitive stress symptoms**	Marginal R2 = 0.29; Conditional R2 = 0.29			Marginal R2 = 0.25; Conditional R2 = 0.25			Marginal R2 = 0.32; Conditional R2 = 0.35			Marginal R2 = 0.30; Conditional R2 = 0.30		
	Beta (std.)	SE*	P*	Beta (std.)	SE*	P*	Beta (std.)	SE*	P*	Beta (std.)	SE*	P*
(Intercept)	0.00	2.65	0.000	0.00	4.22	0.000	0.00	6.06	0.000	0.00	5.04	0.000
Age (years)							-0.12	0.04	0.000			
Years of professional experience	-0.12	0.02	0.000	-0.06	0.04	0.002						
Still in training	0.04	0.78	0.008	0.04	0.93	0.020						
Employees in administration and research				0.05	2.58	0.014				-0.04	3.37	0.092
Physicians	-0.08	1.07	0.000	-0.08	1.44	0.000						
Nurse assistants without formal education	-0.03	2.57	0.016									
Nurse assistants with a formal education	0.03	0.88	0.074									
Registered nurses				-0.05	0.87	0.044	-0.04	1.06	0.062	-0.07	0.90	0.004
Working hours per week							-0.04	0.02	0.092			
Gender	-0.08	0.68	0.000	-0.05	0.84	0.008				-0.04	1.93	0.116
Emotional demands at work	-0.04	0.02	0.002	-0.03	0.03	0.134						
Quantitative demands at work	0.03	0.02	0.056				0.06	0.03	0.036	0.09	0.03	0.000
Demands to hide emotions	0.08	0.01	0.000	0.05	0.02	0.018	0.10	0.02	0.000			
Meaning of work	-0.05	0.02	0.004	-0.07	0.03	0.006	-0.06	0.03	0.014	-0.05	0.04	0.078
Influence at work				-0.05	0.03	0.056						
Scope for breaks/holidays				0.06	0.02	0.004						
Bond with the organization	-0.05	0.02	0.000									
Feedback	-0.04	0.01	0.006									
Quality of leadership	0.07	0.02	0.000							0.12	0.03	0.000
Social community at work				-0.04	0.03	0.068	-0.04	0.03	0.106	-0.06	0.04	0.046
Role clarity	-0.10	0.02	0.000	-0.12	0.03	0.000	-0.10	0.03	0.000	-0.08	0.04	0.008
Role conflicts	0.06	0.02	0.000				0.08	0.02	0.004	0.05	0.03	0.086
Social relations at work				0.05	0.02	0.026						
Unfair behavior	0.05	0.01	0.002	0.04	0.02	0.048						
Social support at work							0.04	0.03	0.184	-0.04	0.03	0.150
Predictability				-0.04	0.03	0.116						
Rewards				0.05	0.02	0.054				-0.07	0.03	0.032
Insecurity of the working environment	0.06	0.01	0.000							0.05	0.02	0.056
Difficulties with demarcation							0.04	0.02	0.132			
Work-private life conflict	0.32	0.02	0.000	0.34	0.02	0.000	0.31	0.03	0.000	0.42	0.03	0.000
Demanding work environment	0.04	0.02	0.010	0.09	0.02	0.000	0.10	0.02	0.000			
Middle-management position							0.04	1.45	0.094			
Upper-management position										-0.03	2.44	0.160
Doing shift work	-0.06	0.71	0.000									
Language region: German-speaking	-0.13	0.87	0.000				-0.15	3.43	0.012			
Language region: French-speaking				0.09	2.97	0.000	-0.06	3.70	0.258			

*based on bootstrapping, Beta (std) = standardized beta coefficients, SE = standard errors

With regard to cognitive stress symptoms, the incompatibility of work and private life was also revealed as a significant predictor among health professionals working in all the included areas (B ≥ 0.31, *p* = 0.000). In addition, a lack of role clarity at work showed a significant association with health professionals’ increased cognitive stress symptoms in all the included areas (B≤-0.08, *p* < 0.01).

### Results of the regression analysis regarding job satisfaction and intention to leave

Further results in Table 4 indicate that good leadership qualities of the direct supervisor were associated with health professionals’ job satisfaction in all the included areas (B ≥ 0.22, *p* = 0.000). Moreover, health professionals’ positive perceived bond with the organization (B ≥ 0.16, *p* = 0.000), social community at work (e.g., atmosphere, co-operation, B ≥ 0.07, *p* = 0.000) and opportunities for development (B ≥ 0.13, *p* = 0.000) were associated with a higher satisfaction at work in all the included areas. The incompatibility of health professionals’ work and private life was shown to be associated with a poorer satisfaction at work in all the included areas (B≤-0.08, *p* = 0.000).

**Table 4:** Work stressors associated with health professionals’ job satisfaction and intention to leave

	Acute care/rehabilitation hospitals			Psychiatric hospitals			Nursing homes			Home care organizations		
**Outcome variable: job satisfaction**	Marginal R2 = 0.68; Conditional R2 = 0.68			Marginal R2 = 0.69, Conditional R2 = 0.69			Marginal R2 = 0.69; Conditional R2 = 0.69			Marginal R2 = 0.65; Conditional R2 = 0.65		
	Beta (std.)	SE*	P*	Beta (std.)	SE*	P*	Beta (std.)	SE*	P*	Beta (std.)	SE*	P*
(Intercept)	0.00	1.53	0.000	0.00	0.00	0.388	0.00	2.35	0.000	0.00	2.60	0.000
Years of professional experience				0.04	0.02	0.004	0.03	0.02	0.052	0.10	0.02	0.000
Years in current position	0.03	0.02	0.004									
Still in training										0.03	0.56	0.080
Employees in administration and research							-0.04	3.23	0.004			
Physicians	0.08	0.57	0.000	0.02	0.73	0.116						
Midwives	-0.02	0.88	0.096									
Nurse assistants with a formal education										0.03	0.52	0.104
Registered nurses	0.02	0.29	0.050									
Working hours per week										0.05	0.01	0.014
Emotional demands at work	-0.03	0.01	0.002				-0.06	0.02	0.000	-0.08	0.02	0.000
Physical demands at work	-0.07	0.01	0.000	-0.09	0.02	0.000	-0.07	0.01	0.000	-0.08	0.01	0.000
Quantitative demands at work	-0.04	0.01	0.000	-0.03	0.01	0.066	-0.07	0.01	0.000	-0.03	0.02	0.112
Demands to hide emotions	-0.04	0.01	0.000				-0.06	0.01	0.000			
Meaning of work	0.05	0.01	0.000	0.03	0.01	0.082	0.06	0.02	0.000			
Influence at work	0.03	0.01	0.002	0.07	0.01	0.000						
Opportunities for development	0.13	0.01	0.000	0.15	0.02	0.000	0.17	0.02	0.000	0.20	0.02	0.000
Bond with the organization	0.19	0.01	0.000	0.16	0.01	0.000	0.18	0.01	0.000	0.22	0.01	0.000
Feedback	0.04	0.01	0.000	0.04	0.01	0.006	0.06	0.01	0.000	0.05	0.01	0.016
Quality of leadership	0.23	0.01	0.000	0.25	0.01	0.000	0.22	0.01	0.000	0.22	0.02	0.000
Social community at work	0.14	0.01	0.000	0.11	0.01	0.000	0.11	0.02	0.000	0.07	0.02	0.000
Social relations at work							0.05	0.01	0.000			
Role clarity				0.03	0.02	0.050	0.03	0.02	0.068			
Role conflicts	-0.05	0.01	0.000	-0.11	0.01	0.000	-0.05	0.01	0.006	-0.03	0.01	0.130
Social support at work	0.04	0.01	0.002	0.03	0.02	0.066				0.05	0.02	0.012
Unfair behavior	-0.04	0.01	0.002	-0.07	0.01	0.000	-0.06	0.01	0.000	-0.03	0.01	0.088
Predictability	0.05	0.01	0.000	0.06	0.01	0.002	0.04	0.01	0.028	0.06	0.02	0.002
Rewards	0.06	0.01	0.000	0.07	0.01	0.000				0.06	0.01	0.006
Insecurity of the working environment	-0.03	0.01	0.006									
Difficulties with demarcation							0.03	0.01	0.042	0.04	0.01	0.016
Work–private life conflict	-0.13	0.01	0.000	-0.08	0.01	0.000	-0.14	0.01	0.000	-0.14	0.02	0.000
Demanding work environment	-0.02	0.01	0.050							-0.04	0.02	0.052
Middle-management position	0.03	0.43	0.000	0.05	0.56	0.000	0.06	0.66	0.002			
Doing shift work				0.04	0.45	0.000				-0.05	0.49	0.010
Language region: German-speaking	0.03	0.49	0.022									
**Outcome variable: intention to leave the organization**	Marginal R2 = 0.36; Conditional R2 = 0.36			Marginal R2 = 0.36; Conditional R2 = 0.36			Marginal R2 = 0.39; Conditional R2 = 0.39			Marginal R2 = 0.30; Conditional R2 = 0.31		
	Beta (std.)	SE*	P*	Beta (std.)	SE*	P*	Beta (std.)	SE*	P*	Beta (std.)	SE*	P*
(Intercept)	0.00	3.63	0.000	0.00	0.00	0.083	0.00	5.44	0.000	0.00	5.26	0.000
Age (years)	-0.14	0.05	0.000	-0.11	0.03	0.003	-0.08	0.05	0.008	-0.10	0.06	0.000
Years of professional experience	-0.04	0.06	0.134	-0.05	0.03	0.125	-0.06	0.06	0.030	-0.07	0.07	0.064
Years in current position				-0.05	0.02	0.022						
Still in training	-0.04	0.86	0.002	-0.04	0.02	0.045	-0.08	1.28	0.000	-0.08	1.22	0.000
Physicians	-0.05	1.26	0.000				-0.04	7.87	0.064			
Nurse assistants without formal education				-0.04	0.02	0.011	-0.08	1.58	0.002	-0.05	1.79	0.066
Nurse assistants with a formal education				-0.09	0.02	0.001	-0.06	1.16	0.002			
Registered nurses	0.05	0.65	0.000							0.05	1.19	0.064
Gender	0.05	0.83	0.000									
Emotional demands at work	0.02	0.02	0.158							0.06	0.05	0.048
Physical demands at work							0.04	0.02	0.078	0.06	0.03	0.032
Quantitative demands at work	0.05	0.02	0.002	0.03	0.02	0.163	0.04	0.03	0.130			
Demands to hide emotions	0.03	0.01	0.060				0.04	0.02	0.126			
Meaning of work	-0.04	0.02	0.006	-0.05	0.02	0.058						
Influence at work							0.03	0.03	0.092	-0.09	0.03	0.000
Opportunities for development	-0.08	0.03	0.000	-0.06	0.02	0.021	-0.09	0.04	0.000	-0.11	0.04	0.000
Bond with the organization	-0.21	0.02	0.000	-0.22	0.02	0.001	-0.25	0.03	0.000	-0.13	0.03	0.000
Quality of leadership	-0.16	0.02	0.000	-0.16	0.02	0.001	-0.14	0.03	0.000	-0.10	0.04	0.008
Social community at work	-0.03	0.02	0.014				-0.04	0.04	0.082			
Role conflicts	0.07	0.02	0.000	0.05	0.02	0.020	0.07	0.03	0.004	0.09	0.03	0.004
Social support at work							0.04	0.04	0.150			
Unfair behavior	0.05	0.02	0.002	0.06	0.02	0.010	0.07	0.02	0.004	0.07	0.03	0.000
Predictability	0.03	0.02	0.080									
Rewards	-0.04	0.02	0.038	-0.05	0.02	0.037				-0.04	0.03	0.154
Insecurity of the working environment				0.03	0.02	0.102				-0.05	0.03	0.062
Difficulties with demarcation	0.03	0.01	0.050									
Work–private life conflict	0.18	0.02	0.000	0.18	0.02	0.000	0.19	0.03	0.000	0.17	0.03	0.000
Middle-management position	0.02	0.96	0.126	0.03	0.02	0.148				0.05	1.75	0.028
Upper-management position										0.04	2.74	0.104
Doing shift work	-0.04	0.81	0.002									
Language region: German-speaking	0.04	0.89	0.006	0.07	0.02	0.000	0.06	1.41	0.012			
**Outcome variable: intention to leave the profession**	Marginal R2 = 0.29; Conditional R2 = 0.29			Marginal R2 = 0.27; Conditional R2 = 0.28			Marginal R2 = 0.36; Conditional R2 = 0.37			Marginal R2 = 0.26; Conditional R2 = 0.27		
	Beta (std.)	SE*	P*	Beta (std.)		Beta (std.)	SE*	P*	Beta (std.)		Beta (std.)	SE*
(Intercept)	0.00	3.18	0.000	0.00	5.12	0.000	0.00	6.10	0.000	0.00	6.27	0.000
Age (years)	-0.12	0.03	0.000	-0.09	0.04	0.000	-0.09	0.04	0.000	-0.13	0.05	0.000
Still in training	-0.02	0.84	0.116				-0.04	1.27	0.090	-0.10	1.25	0.000
Employees in administration and research	0.02	1.77	0.074							-0.05	3.82	0.044
Physicians	-0.04	1.31	0.004	-0.04	1.65	0.096						
Nurse assistants without formal education				-0.03	3.28	0.080	-0.08	1.57	0.002			
Nurse assistants with a formal education	0.07	1.03	0.000				-0.06	1.40	0.050			
Registered nurses	0.04	0.66	0.006	0.04	1.01	0.072	-0.06	1.47	0.032	0.04	1.11	0.210
Working hours per week	-0.03	0.01	0.048				-0.04	0.02	0.106			
Physical demands at work	0.04	0.02	0.006				0.04	0.02	0.086	0.06	0.03	0.036
Emotional demands at work										0.05	0.05	0.086
Quantitative demands at work	0.04	0.02	0.008									
Demands to hide emotions	0.06	0.01	0.000	0.06	0.02	0.012	0.07	0.02	0.012	0.05	0.02	0.102
Meaning of work	-0.11	0.02	0.000	-0.11	0.03	0.000	-0.04	0.04	0.062	-0.07	0.04	0.016
Influence at work										-0.06	0.03	0.028
Opportunities for development	-0.05	0.02	0.002	-0.07	0.03	0.004	-0.05	0.04	0.024	-0.13	0.04	0.000
Scope for breaks/holidays				-0.04	0.02	0.096	0.03	0.02	0.164			
Bond with the organization	-0.18	0.02	0.000	-0.17	0.03	0.000	-0.22	0.03	0.000	-0.11	0.03	0.002
Quality of leadership	-0.03	0.02	0.106	-0.07	0.03	0.010						
Social community at work				-0.03	0.03	0.104	-0.04	0.03	0.048			
Role conflicts	0.05	0.02	0.004	0.04	0.03	0.100	0.05	0.03	0.062	0.08	0.03	0.006
Social relations at work				0.04	0.02	0.104						
Social support at work							0.04	0.03	0.086			
Unfair behavior	0.05	0.01	0.000	0.05	0.02	0.034				0.07	0.03	0.004
Predictability	0.04	0.02	0.000							0.06	0.03	0.038
Rewards	-0.05	0.01	0.008	0.03	0.02	0.168						
Insecurity of the working environment	0.03	0.01	0.048				0.08	0.02	0.000			
Work–private life conflict	0.23	0.02	0.000	0.19	0.03	0.000	0.31	0.03	0.000	0.21	0.03	0.000
Demanding work environment				0.06	0.03	0.010				0.05	0.03	0.056
Gender										0.08	2.22	0.002
Upper-management position				0.03	1.90	0.124						
Doing shift work				-0.04	1.08	0.094				-0.06	1.12	0.016
Language region: German-speaking	0.07	1.02	0.000	0.03	2.53	0.248	0.12	1.39	0.000			

*based on bootstrapping, Beta (std) = standardized beta coefficients, SE = standard errors

In addition, the results showed that a poor perceived compatibility of work and private life (B ≥ 0.17, *p* = 0.000), bond with the organization (B≤-0.13, *p* < 0.01), quality of leadership (B≤-0.10, *p* < 0.01), and opportunities for development (B≤-0.06, *p* < 0.01) were associated with health professionals’ higher intention to leave the organization in all the included areas. Furthermore, this higher intention to leave the organization was also associated with higher role conflicts (e.g., due to contradicting role requirements at work, B ≥ 0.05, *p* < 0.05) and a perceived unfair behavior (e.g., feeling unjustly criticized by colleagues/superior, B ≥ 0.05, *p* < 0.05) in all the included work areas.

The incompatibility of work and private life (B ≥ 0.19, *p* = 0.000), a poor perceived bond with the organization (B≤-0.11, *p* < 0.01) and a lack of opportunities for development (B≤-0.05, *p* < 0.05) were also associated with health professionals’ higher intention to leave the profession prematurely in all the included work areas. For health professionals working in hospitals and home care organizations, a lower meaning of work (B≤-0.07, *p* < 0.05) was also found to be a significant predictor for a higher intention to leave their profession prematurely. Moreover, further results showed that the younger the age of the health professionals, the higher their intention to leave the organization (B≤-0.08, *p* < 0.01) and their profession prematurely (B≤-0.09, *p* = 0.000) in all the included work areas.

### Results of the regression analysis regarding health-related outcomes

The results in Table 5 on health professionals’ health-related outcomes revealed an incompatibility of work and private life (B≤-0.24, *p* < 0.01) as well as increased physical demands at work (e.g., lifting or moving people or heavy loads, B≤-0.09, *p* < 0.01) were associated with a poorer general health status among health professionals working in all the included areas. Physicians working in acute care, rehabilitation, and psychiatric hospitals reported a higher general health status (B ≥ 0.05, *p* < 0.05).

**Table 5:** Work stressors associated with health professionals’ health-related outcomes

	Acute care/rehabilitation hospitals			Psychiatric hospitals			Nursing homes			Home care organizations		
**Outcome variable: general health status**	Marginal R2 = 0.22; Conditional R2 = 0.22			Marginal R2 = 0.22, Conditional R2 = 0.21			Marginal R2 = 0.23; Conditional R2 = 0.25			Marginal R2 = 0.17; Conditional R2 = 0.20		
	Beta (std.)	SE*	P*	Beta (std.)	SE*	P*	Beta (std.)	SE*	P*	Beta (std.)	SE*	P*
(Intercept)	0.00	3.00	0.000	0.00	0.00	0.496	0.00	3.84	0.000	0.00	4.58	0.000
Age (years)	-0.03	0.02	0.076	-0.04	0.02	0.076				-0.09	0.05	0.012
Years of professional experience										0.05	0.05	0.152
Employees in administration and research				-0.06	0.02	0.004						
Physicians	0.07	1.11	0.000	0.05	0.02	0.019	0.04	6.50	0.092			
Nurse assistants with a formal education	-0.05	0.94	0.000				0.06	1.03	0.030			
Registered nurses	0.04	0.56	0.034				0.05	1.13	0.040			
Gender	0.02	0.68	0.138	-0.03	0.02	0.125						
Physical demands at work	-0.09	0.01	0.000	-0.10	0.02	0.001	-0.13	0.02	0.000	-0.14	0.03	0.000
Quantitative demands at work							0.07	0.03	0.016	-0.05	0.03	0.134
Demands to hide emotions	-0.05	0.01	0.002									
Meaning of work	0.05	0.02	0.006	0.06	0.03	0.02						
Influence at work	-0.03	0.01	0.106									
Opportunities for development	0.06	0.02	0.006				0.04	0.03	0.144	0.09	0.03	0.014
Scope for breaks/holidays	0.05	0.01	0.000							-0.05	0.02	0.120
Bond with the organization	0.05	0.02	0.004	0.05	0.03	0.077	0.09	0.02	0.000	0.05	0.03	0.088
Feedback				0.05	0.02	0.032				-0.04	0.03	0.196
Quality of leadership	-0.03	0.02	0.100				-0.04	0.03	0.176	-0.07	0.03	0.052
Social community at work	0.03	0.02	0.094				0.09	0.03	0.000	0.05	0.04	0.050
Role clarity				0.07	0.03	0.006	0.04	0.03	0.172			
Role conflicts				-0.04	0.03	0.103						
Social support at work	0.03	0.02	0.168									
Unfair behavior	-0.04	0.01	0.006	-0.05	0.02	0.036						
Predictability							0.04	0.03	0.230			
Rewards	0.04	0.01	0.046							0.11	0.02	0.000
Insecurity of the working environment	-0.04	0.01	0.028							-0.07	0.02	0.046
Difficulties with demarcation	-0.02	0.01	0.116	-0.05	0.02	0.037	-0.09	0.02	0.004			
Work–private life conflict	-0.27	0.01	0.000	-0.28	0.03	0.001	-0.27	0.02	0.000	-0.24	0.03	0.000
Demanding work environment							-0.07	0.02	0.006			
Middle-management position				0.04	0.02	0.094						
Doing shift work										0.07	0.94	0.014
Language region: German-speaking	0.03	0.76	0.058									
Language region: French-speaking				-0.05	0.02	0.033	-0.04	1.51	0.200			
**Outcome variable: burnout symptoms**	Marginal R2 = 0.40; Conditional R2 = 0.40			Marginal R2 = 0.36; Conditional R2 = 0.36			Marginal R2 = 0.40; Conditional R2 = 0.42			Marginal R2 = 0.38; Conditional R2 = 0.39		
	Beta (std.)	SE*	P*	Beta (std.)	SE*	P*	Beta (std.)	SE*	P*	Beta (std.)	SE*	P*
(Intercept)	0.00	2.48	0.000	0.00	4.18	0.000	0.00	4.92	0.000	0.00	4.01	0.000
Age (years)	-0.12	0.04	0.000	-0.10	0.03	0.000	-0.12	0.03	0.000			
Years of professional experience	-0.06	0.04	0.018							-0.12	0.04	0.000
Still in training										-0.03	1.12	0.266
Physicians	-0.03	1.05	0.018	-0.05	1.38	0.002						
Midwives	0.03	1.53	0.014									
Nurse assistants with a formal education	0.04	0.83	0.000				-0.04	1.10	0.140	0.06	1.08	0.014
Registered nurses				-0.04	0.89	0.052	-0.06	1.24	0.014			
Working hours per week										-0.04	0.02	0.126
Gender	-0.07	0.70	0.000	-0.07	0.80	0.000	-0.07	1.32	0.002	-0.07	2.04	0.004
Emotional demands at work				0.04	0.03	0.042	0.07	0.03	0.000	0.07	0.04	0.010
Physical demands at work	0.04	0.01	0.016	0.06	0.03	0.004				0.08	0.03	0.002
Quantitative demands at work	0.05	0.02	0.000				0.08	0.03	0.000	0.14	0.03	0.000
Demands to hide emotions	0.05	0.01	0.002	0.04	0.02	0.030	0.13	0.02	0.000	0.06	0.02	0.012
Meaning of work	-0.05	0.02	0.000	-0.04	0.02	0.052						
Influence at work	-0.03	0.01	0.044									
Bond with the organization	-0.06	0.02	0.000	-0.05	0.02	0.010	-0.08	0.03	0.000	-0.06	0.03	0.004
Feedback	-0.02	0.01	0.114	-0.04	0.02	0.038	-0.05	0.02	0.040			
Social community at work							-0.05	0.03	0.040			
Role clarity							-0.05	0.03	0.024			
Role conflicts	0.04	0.02	0.010	0.05	0.02	0.040				0.04	0.03	0.068
Social support at work							0.05	0.03	0.042			
Unfair behavior	0.04	0.01	0.006									
Rewards	-0.02	0.01	0.184									
Insecurity of the working environment	0.05	0.01	0.000							0.04	0.02	0.128
Work–private life conflict	0.39	0.02	0.000	0.46	0.02	0.000	0.38	0.02	0.000	0.41	0.03	0.000
Demanding work environment	0.05	0.02	0.000	0.04	0.02	0.044	0.08	0.03	0.000			
Middle-management position							0.04	1.50	0.140			
Upper-management position	-0.02	1.20	0.088	0.03	1.69	0.130						
Doing shift work	-0.08	0.69	0.000	-0.06	0.90	0.008						
Language region: German-speaking	-0.10	0.83	0.000	0.07	2.47	0.016						
Language region: French-speaking				0.11	3.83	0.000	0.07	1.69	0.006			
**Outcome variable: quality of sleep**	Marginal R2 = 0.24; Conditional R2 = 0.24			Marginal R2 = 0.23; Conditional R2 = 0.23			Marginal R2 = 0.29; Conditional R2 = 0.30			Marginal R2 = 0.20; Conditional R2 = 0.25		
	Beta (std.)	SE*	P*	Beta (std.)	SE*	P*	Beta (std.)	SE*	P*	Beta (std.)	SE*	P*
(Intercept)	0.00	2.56	0.000	0.00	0.00	0.390	0.00	5.62	0.000	0.00	5.14	0.000
Age (years)							0.12	0.04	0.000			
Years in current position	0.03	0.03	0.056							0.04	0.07	0.106
Years of professional experience							-0.10	0.05	0.000			
Midwives	-0.02	1.68	0.174									
Employees in administration and research							0.05	6.54	0.048			
Physicians	0.04	1.06	0.002	0.04	0.02	0.100						
Nurse assistants without formal education	0.02	2.53	0.160									
Nurse assistants with a formal education	-0.03	0.84	0.034	-0.06	0.02	0.003	0.05	0.94	0.042	-0.05	1.06	0.056
Working hours per week	0.11	0.01	0.000	0.11	0.02	0.000	0.04	0.02	0.098	0.07	0.02	0.006
Emotional demands at work	-0.04	0.02	0.000				-0.07	0.03	0.000	-0.04	0.04	0.180
Physical demands at work							-0.05	0.02	0.046	-0.04	0.03	0.100
Quantitative demands at work				0.03	0.02	0.119						
Demands to hide emotions										-0.04	0.02	0.126
Meaning of work				0.06	0.02	0.005	0.06	0.03	0.022	0.06	0.04	0.050
Opportunities for development	0.07	0.02	0.000				0.05	0.03	0.056	0.07	0.04	0.016
Bond with the organization	0.05	0.01	0.002							-0.04	0.03	0.182
Quality of leadership	-0.04	0.02	0.038									
Social community at work	0.04	0.02	0.016	0.05	0.02	0.019						
Social relations at work				-0.05	0.02	0.021						
Role clarity										-0.03	0.04	0.354
Role conflicts	-0.04	0.02	0.008									
Social support at work	0.04	0.02	0.034				0.05	0.03	0.032	0.05	0.03	0.134
Predictability										0.04	0.03	0.278
Rewards				0.04	0.02	0.100						
Insecurity of the working environment	-0.06	0.01	0.000	-0.07	0.02	0.007	-0.06	0.02	0.050	-0.06	0.03	0.036
Difficulties with demarcation	-0.05	0.01	0.000	-0.05	0.02	0.015				-0.05	0.02	0.042
Work–private life conflict	-0.31	0.02	0.000	-0.36	0.03	0.001	-0.34	0.02	0.000	-0.31	0.03	0.000
Demanding work environment	-0.05	0.02	0.004	0.06	0.02	0.006	-0.05	0.03	0.042			
Middle-management position							0.08	1.43	0.004			
Upper-management position							-0.03	2.98	0.182			
Language region: German-speaking	0.15	0.75	0.000	0.10	0.02	0.000	0.21	2.76	0.000			
Language region: French-speaking							0.09	2.89	0.052			

*based on bootstrapping, Beta (std) = standardized beta coefficients, SE = standard errors

In addition, incompatibility between work and private life (B ≥ 0.38, *p* = 0.000) was associated with health professionals’ increased burnout-symptoms in all the included areas. Furthermore, high quantitative (B ≥ 0.05, *p* = 0.000) and physical (B ≥ 0.04, *p* < 0.05) demands at work were revealed as significant predictors for increased burnout symptoms among health professionals working in various areas. Further results showed that the younger the age, the higher the burnout symptoms (B≤-0.10, *p* = 0.000) for health professionals’ working in hospitals and nursing homes.

The incompatibility of health professionals’ work and private life was also significantly associated with a poor quality of sleep (B≤-0.31, *p* < 0.01) in all the included areas. In addition, a higher insecurity of the working environment (e.g., changes in shift schedules, B≤-0.06, *p* < 0.05) and difficulties with demarcation (e.g., being available in leisure time for work issues, B≤-0.05, *p* < 0.05) were associated with a poorer quality of sleep for health professionals working in hospitals and home care organizations. A demanding work environment (e.g., being exposed to noise, cold, chemicals, B≤-0.05, *p* < 0.05) was also significantly associated with health professionals’ poorer quality of sleep among those working in hospitals and nursing homes. Health professionals’ higher perceived meaning of work was associated with a better quality of sleep (B ≥ 0.06, *p* < 0.05) among those working in psychiatric hospitals and nursing homes. Further results relating to the specific field of work of health professionals are shown in Tables 3, 4 and 5.

**Fig. 2:**
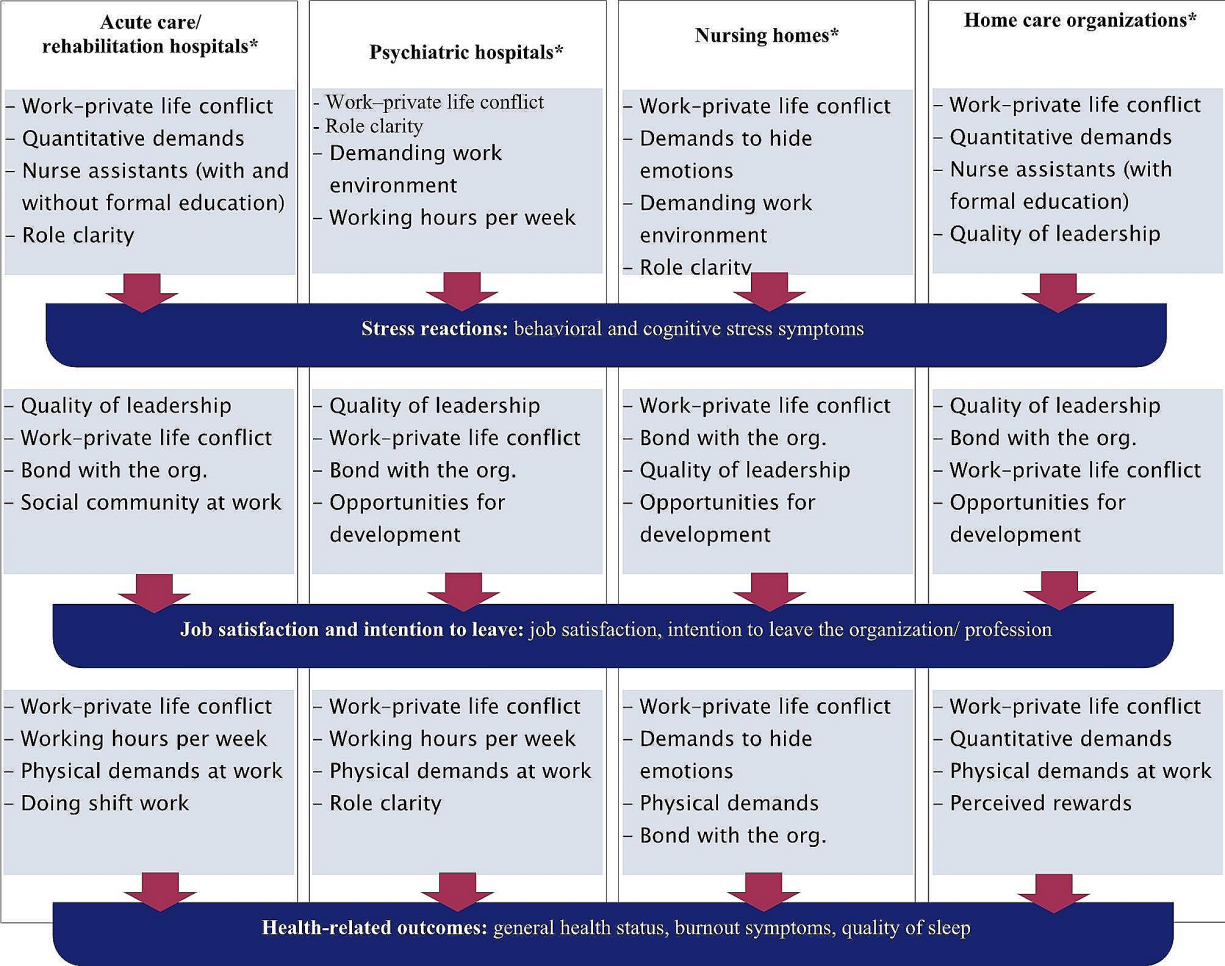
Stressors at work associated with health professionals’ stress symptoms, job satisfaction, intention to leave and health-related outcomes working in various areas (top 4 significant predictors regarding standardized Beta coefficients)

Figure 2 presents an overview of the top four significant predictors of health professionals’ stress reactions, job satisfaction, intention to leave, and health-related outcomes for acute care / rehabilitation hospitals, psychiatric hospitals, nursing homes, and home care organizations.

## Discussion

This study presents, for the first time, detailed results for health professionals working in different work areas in Switzerland and enables a direct comparison of working conditions among hospitals, nursing homes, and home care organizations. The results of this study indicate a higher extent of various stressors (e.g., higher quantitative, sensorial demands, role conflicts, and work-private life conflicts, lower influence at work, rewards, feedback, and quality of leadership), stress reactions (higher cognitive stress symptoms) and long-term consequences (e.g., lower job satisfaction and quality of sleep, higher intention to leave and burnout-symptoms) among health professionals working in acute care and rehabilitation hospitals (compared to other sectors). However, there are also relevant stressors regarding other work areas (e.g., high emotional and physical demands in nursing homes, the lower meaning of work and role clarity in psychiatric hospitals, lower feedback and social relations at work in home care organisations). This appears to be related to the specific role, job content and responsibilities of health professionals, their different working environments and the nature of patient care in these different areas of work [4, 41].

Further results of this study show that the incompatibility of work and private life is one of the most important predictors for health professionals’ increased stress reactions, job dissatisfaction, higher intention to leave the organization and the profession, as well as negative health-related outcomes in acute care, rehabilitation, and psychiatric hospitals, nursing homes, and home care organizations, which is in line with prior literature [4, 16, 18, 19]. A previous literature review including various EU and non-EU studies identified job satisfaction, work–life balance and career development as the main determinants for health professionals’ job retention [42]. A lack of opportunities for development was also associated with health professionals’ lower job satisfaction and greater intention to leave the organization and their profession in all the included work areas in this study.

Moreover, further results of this study revealed that good leadership qualities of the direct supervisor are also significantly associated with health professionals’ satisfaction at work in all the work areas included in this study. Previous studies indicate that health professional leaders have an important role regarding work-related stress among their employees [16, 43]. As the results of a previous review on leadership and health professionals’ job satisfaction conclude, it is important to identify and close the gaps in leadership knowledge and for leaders to play a key role in improving health professionals’ satisfaction at work [44].

Another important result of this study revealed that the intention to leave the organization and the profession prematurely was associated with a younger age of health professionals working in all of the included work areas. In addition, a younger age was also significantly associated with increased burnout symptoms for those working in acute care, rehabilitation, and psychiatric hospitals, and nursing homes. These results are essential regarding the shortage of health professionals in the future [1, 45]. Previous study results indicate that newly graduated health professionals are at risk of leaving their profession prematurely and also determined increased burnout symptoms [46, 47]. Especially during their first year of practice, 60–74% of newly graduated nurses showed the intention to leave their profession right away [48–50]. Several studies identified stressors at work (high quantitative demands, working overtime, work–private life conflicts), insufficient induction and support from colleagues and leaders, unfulfilled expectations along with a lack of support in finding one’s own role as possible causes [46, 47, 50, 51]. However, for an effective job retention of health professionals and an adequate future staffing in the healthcare sector, better support of young health professionals is essential. Therefore, evidence-based and effective programs to support newly graduated health professionals during their transition phase into daily practice are important in order to retain them for the long-term in the healthcare sector [52, 53].

### Strengths and limitations

These results differ mainly from those of other studies because they are based on a large sample of health professionals (including nurses, midwives, physicians, medical-technical, and medical-therapeutic professionals) working in different work areas (acute care, rehabilitation, psychiatric hospitals, nursing homes, home care organizations) among different language regions (German, French, Italian). Moreover, the results of this study rely on several data measurements, using well established, valid, and reliable scales to assess work stressors, stress reactions, and long-term consequences.

However, participation in the study was on a voluntary basis for all the invited organizations and health professionals, and so a selection bias cannot be excluded (e.g., whether health professionals with higher levels of work-related stress did not participate due to restricted time resources). Also, nurses dominated the study sample in nursing homes and home care organizations, while in acute care, rehabilitation, and psychiatric hospitals a greater variation of health professions was represented. In addition, the third data measurement (T2) was conducted during the coronavirus pandemic, which could have had a negative impact on the willingness to participate by health professionals and also on their self-reports with regard to stressors and consequences at work. The generalisability of the results may be limited, as nurses and midwives are slightly overrepresented in the study sample.

## Conclusions

Strategies for practice organisations, shaped to their specific working conditions regarding salient stressors in their area of work, are important (e.g., reducing workload and work-private live conflicts among health professionals in acute care and rehabilitation hospitals, stronger emotional support and use of patient lifter to reduce physical demands in nursing homes, improving role clarity in psychiatric hospitals and improving information transfer and team communication in home care organisations.

In addition, the importance of a good work–life balance, actively managing staff career development, and fostering staff commitment to their organization have emerged as key topics for healthcare organizations in all sectors to keep their staff healthy and satisfied in the long term. Furthermore, as the results of this study indicate, leaders should be aware of the most relevant stressors in their work area. While there are common stressors (e.g. work–private life conflicts, lack of opportunities for development), there are also differences across settings (as presented in Fig. 2). As our results show, it is also important to pay special attention to young health professionals in order to keep them in the healthcare system long-term and are not lost as soon as they enter the workforce. Therefore, evidence-based, effective, and interprofessional programs are important in order to better support young health professionals in dealing with stressors at work and finding their role during their transition phase. On the one hand, health organizations as employers are in demand, and on the other hand, the educational organizations that train future health professionals.

## Data Availability

The raw data set analyzed in the current study is available from the corresponding author on reasonable request.
